# Gear Tooth Profile Reconstruction via Geometrically Compensated Laser Triangulation Measurements

**DOI:** 10.3390/s19071589

**Published:** 2019-04-02

**Authors:** Hao Tian, Fan Wu, Yongjun Gong

**Affiliations:** Department of Mechanical Engineering, Dalian Maritime University, Dalian 116026, China; wufan@dlmu.edu.cn

**Keywords:** laser triangulation sensors, displacement sensing, gear tooth profile reconstruction

## Abstract

Precision modeling of the hydraulic gear pump pressure dynamics depends on the accurate prediction of volumetric displacement in the inter-tooth spaces of the gear. By accurate reconstruction of the gear profile, detailed transient volumetric information can be determined. Therefore, this paper reports a non-contact gear measurement device using two opposing laser triangulation sensors, and the key geometrical models to reconstruct the profile with geometrical error compensation. An optimization-based key parameter calculation method is also proposed to find the unknown orientation of the sensor. Finally, an experimental setup is established, the performance of the device is tested and the geometric model is validated. Initial results showed that the method is able to reconstruct the target tooth profile and compensated results can reduce the geometrical error by up to 98% compared to the uncalibrated ones.

## 1. Introduction

### 1.1. Background

Gears are not only widely used in transmission of mechanical power, but are key mechanical elements found in the hydraulic gear pumps, where the meshing gear and pinon pairs carry the hydraulic oil in the fixed-size inter-tooth space, delivering the pressurized oil. For a gear pump or any positive displacement fluid power unit, the dynamic pressure within the component is determined by the transient volumetric and leakage properties of the control volume [1]. In contrast to traditional models of a gear pump or motor, which are based on the ideal involute or cycloidal gear profiles, the state-of-the-art ones often take the detailed tooth profile into account, resulting in more accurate prediction of volumetric variation or leakage of fluid within the inter-tooth space [2,3,4,5,6]. Thus, accurate reconstruction of gear tooth profile functions in three-dimensional (3D) space rather than obtaining key summarizing parameters can lead to better prediction of friction and leakage characteristics of a hydraulic gear pump or motor.

Traditional gear tooth profile measurement methods can be classified into the contact or noncontact approaches based on whether the measuring device is applying force to the target gear. Contact methods employ a stylus or shaped probe as the displacement sensing gauge, driven by a coordinate measuring machine (CMM) [7,8]. By thresholding the contact force on the surface of the gear tooth at a number of discrete locations, the geometric error and key gear properties can be obtained via curve fitting techniques [7,8,9,10,11]. Normally the measurement targets are the power transmitting gears in heavy machineries, and the 3D CAD files for the target gears are required in advance [8,10]. One challenge in contact measuring is the compensation of the pseudo tooth profile deviations induced by the contact stress and the compliance of the stylus based on elastic theory [12]. Another is the accurate interpolation of the discrete probed points of a complex feature based on novel algorithm or numerical optimization [13,14]. In contrast, the gears in mobile hydraulic machineries are comparatively small (e.g., the pitch diameter is on the order of a few centimeters) in size due to the compactness of fluid power component. Some off-the-shelf models would even have extensive modifications to the gear geometry that no prior CAD files or standards are available, rendering measurement of the complete tooth profile with the contact approach a challenge. Especially, the stylus diameter cannot infinitely reduce until the structural compliance becomes too great to be predicted with linear elastic theories, and the tight opening could also pose a mechanical obstacle for the probing devices. Noncontact methods use a laser triangulation sensor (LTS) or digital camera(s) to perform the measurement directly [15,16,17,18]. Without a physical probe, the contact stress, structural deformation, and friction problems are automatically resolved. It is theoretically possible for the noncontact method to profile the gear tooth continuously and quickly without the need for precision contact stress control. But the challenges are still present in the design of the scanning device, calibration, and the postprocessing of data [19]. Therefore, the motivation of this work is to develop a method to reconstruct the profiles for the gear elements found in fluid power systems in particular. The goal is to design and analyze a new noncontact gear tooth profile measurement apparatus, capable of measuring unknown gear profile through a single pass of scan, with the primary focus on the sensor and target placement error compensation.

### 1.2. Principle of Gear Profile Measurement Apparatus

**Figure 1:**
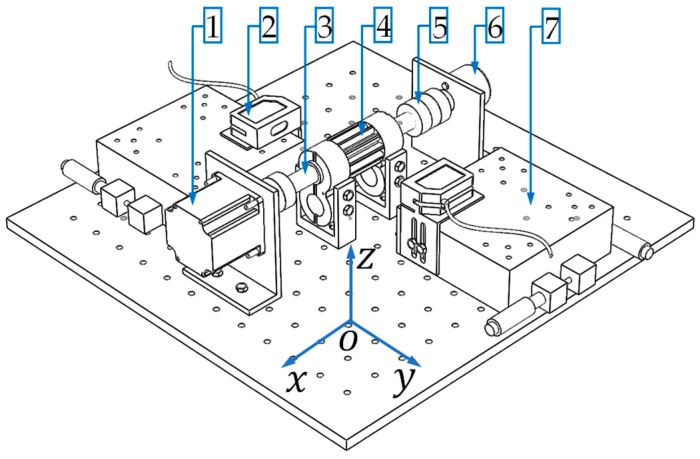
The proposed apparatus to measure the gear tooth profiles: **1**: Stepper motor; **2**: laser triangulation sensor (LTS); **3**: gear shaft; **4**: target gear and a pair of supporting journal bearings; **5**: flexible shaft coupler; **6**: rotary encoder; and **7**: micro motion stage.

A schematic diagram of the proposed gear profile measurement apparatus is shown in Figure 1. There are three key modules: (1) the mechanical system to mount the target gear, (2) the LTS and the supporting stage, and (3) the motion control and signal processing unit. The working principle is described as follows. The target gear is mounted on a shouldered shaft, supported by a pair of precision journal bearings. In this way, the only degree of freedom permitted is the revolution about the gear rotational axis. The surface displacement of the gear tooth is measured using two LTS’s facing each other towards the gear axis. Each of the LTS is mounted on a micro motion stage, where the two prismatic sliding joints permit translation along the *x* or *y* axes and the working envelop is similar to that of a Cartesian robot. The twin LTS setup allows the one sensor to cover the other’s blind spot, enabling single pass measurement of the complete gear tooth profile without the need to reorient the sensor direction contrast to [15], despite the additional requirement of combining the two sensors’ data. The apparatus also includes gear shaft rotational drive, and angular displacement measuring sensors, as well as data acquisition and control. In the test, the micro stages carry the two LTS’s to move in the *y* axis in synchronization, and the controlled rotation of the gear shaft allows complete measurement of the tooth profile.

To implement the proposed gear profile measurement apparatus, two challenges need to be overcome. First, the gear profile needs to be reconstructed from the sensor data based on the geometrical models in 3D space. Second, the geometrical errors, including the mounting error of the gear shaft and the LTS sensor, need to be compensated and calibrated. Thus, the paper is organized as follows. Section 2 focuses on the derivation of the key geometrical models to reconstruct the profile. An optimization-based key parameter calculation method is proposed to find the correct orientations of the LTS’s. A low-cost prototype experimental testbed is setup and the test procedures discussed in Section 3. Finally, 3D reconstruction of a single tooth profile is presented, the measurement error regarding the geometrical disturbances are discussed in Section 4 and Section 5. Initial results demonstrate the validity of the apparatus, and based on the proposed optimization and compensation models, the calibrated profile can reduce the measurement error by up to 98% compared to the uncalibrated ones.

## 2. Geometrical Models

During the tooth profile measurement, the relative position between the LTS and the gear constantly updates as the laser beam is scanning the cross-sections of the gear while the gear is in revolution. Ideally, the LTS’s beam should be positioned normal to the gear’s rotational axis, and the mounting surface of the sensor should be parallel to the rotational axis. However, due to installation error of the LTS’s and the combined geometrical tolerance of both the platforms and the gear shaft support, the actual orientation of the LTS may differ from that of the ideal. It is therefore necessary to first establish a geometrical model to fully describe the mathematical relationship between the measured gear profile and the spatial deviations.

### 2.1. The Vector Relationship between LTS and Tooth Profile

**Figure 2:**
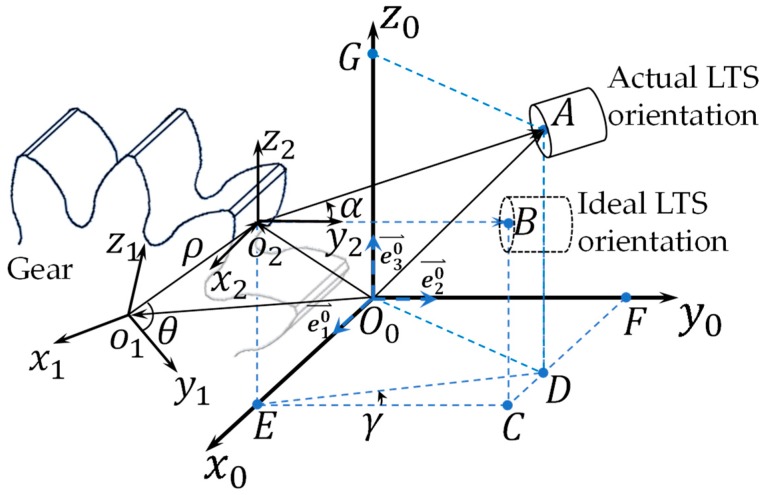
The spatial geometrical relations of the LTS with respect to the target gear, the pitch angle (α) and yaw angle (γ) are exaggerated for illustration. Note the proposed measurement system employs two LTS’s facing opposite directions, only one LTS is shown here for clarity. The other geometrical relationship is the mirrored image about the *x*_1_ axis.

In the measurement of the tooth profile, the goal is to express the tooth profile functions (i.e., radius and angle) in the gear reference frame that the rotational axis of the gear is coincident with one of the reference frames’ axes. To facilitate the analysis, three reference frames are constructed in Figure 2, a cartesian coordinate system is given as *o*_0_*x*_0_*y*_0_*z*_0_, defined as the global reference frame, and the two local reference frames *o*_1_*x*_1_*y*_1_*z*_1_ and *o*_2_*x*_2_*y*_2_*z*_2_, representing the gear and LTS reference frames, which origins are designated at the rotational center of the gear and the contact point of the measuring beam on the surface of the gear, respectively.

Each of the LTS in the proposed system measures the distance between the contact point on the gear tooth and the sensor, which can be represented by a vector pointing from the contact point to the sensor reception window in the sensor reference frame *o*_2_*x*_2_*y*_2_*z*_2_. Under ideal condition, the direction of the laser beam is coincident with the *y*_2_ axis, resulting in the vector ${\vec{p}}_{O_{2}B}$; while the actual orientation of the laser beam could be a pitch about the *x*_2_ axis through an angle of α and a yaw of γ about the z_2_ axis, resulting in ${\vec{p}}_{O_{2}A}$. The roll angle about *y*_2_ axis, however, does not incur variation to the vector, since the rotation of a vector about its axis stays identical. At a given instance of time, the following vector’s identity can be found from the two spatial triangles $\Delta O_{0}O_{2}A$ and $\Delta O_{0}O_{1}O_{2}$:

(1) $${\vec{p}}_{O_{0}O_{1}}={\vec{p}}_{O_{0}A}-{\vec{p}}_{O_{2}A}-{\vec{p}}_{O_{0}O_{1}},$$

where $\vec{p}\in {\mathbb{R}}^{3}$ represents a vector in 3D space, which subscripts denote the origin and destination. The vector ${\vec{p}}_{O_{0}O_{1}}$ denotes the offset between the two origins in the global and gear reference frames, ${\vec{p}}_{O_{0}A}$ defines the geometric relationship of the LTS with respect to the global reference frame, which is updated as the micro motion platform displaces the LTS at discrete positions along the gear rotational axis, and ${\vec{p}}_{O_{2}A}$ is the reoriented vector ${\vec{p}}_{O_{2}B}$.

Particularly, in the coordinate systems defined in Figure 2, the coordinate of point *A* in ${\vec{p}}_{O_{0}A}$ can be found as

(2) $$p_{O_{0}A}^{0}=\left[\begin{array}{c} p_{O_{0}E,j}^{0}\\ p_{O_{0}F,j}^{0}\\ p_{O_{0}G,j}^{0} \end{array}\right],$$

where $p_{O_{0}E,j}^{0}$, $p_{O_{0}F,j}^{0}$, and $p_{O_{0}G,j}^{0}$ represent the measured distances from the measuring center of the LTS to the origin of the global reference frame at the *j*-th LTS, the superscript of the coordinate denotes its reference frame.

Finally, under free vector assumption, the following rotational relationship is found in (3):

(3) $${\vec{p}}_{O_{2}A}=R_{t}{\vec{p}}_{O_{2}B},$$

where the magnitude of ${\vec{p}}_{O_{2}B}$ is obtained experimentally from the readings of the LTS, and the rotation matrix $R_{t}$ is defined in the Section 2.2 and Section 2.3.

### 2.2. Rotation Matrix $R_{t}$

In proposed apparatus, the orientation of the installed LTS could deviate from the ideal one, requiring compensation. To tackle the issue, the rotation of the vector from the ideal orientation to the actual one can be determined by a series of rotations. The order of rotation is specified as follows, first a pitch about the *x*_2_ axis through an angle α, then a roll about the $y_{2}$ axis through β, and a yaw about the *z*_2_ axis through γ. The rotation matrix for the new LTS orientation is then

(4) $$R_{t}=\left[\begin{array}{ccc} \begin{array}{c} \cos\gamma \\ \sin\gamma \\ 0 \end{array} & \begin{array}{c} -\sin\gamma \cos\alpha \\ \cos\gamma \cos\alpha \\ \sin\alpha \end{array} & \begin{array}{c} \sin\gamma \sin\alpha \\ -\cos\gamma \sin\alpha \\ \cos\alpha \end{array} \end{array}\right],$$

The details of the matrix formulation can be found in Appendix A. Particularly, if the LTS orientation is ideal, i.e., the dot product of the respective unit vectors in the frames are unity ($\vec{e_{1}^{0}}\cdot \vec{e_{1}^{1}}=1$, $\vec{e_{2}^{0}}\cdot \vec{e_{2}^{1}}=1$, and $\vec{e_{3}^{0}}\cdot \vec{e_{4}^{1}}=1$), one can simply find $R_{t}=I^{3\times 3}$.

### 2.3. Optimization-Based LTS Orientation Identification

According to Equation (4), there are two unknowns in the displacement measurements using the LTS’s, namely, the pitch angle α, and the yaw angle γ. Since only one parameter can be obtained using the LTS, that is, the displacement from the gear, one cannot directly obtain the pitch and yaw angles. To tackle this issue, an optimization is designed to permutate through a range of pitch and yaw angles until the desired values are acquired for the two LTS’s. The objective function is to find $\alpha_{k}$ and $\gamma_{m}$ that satisfies the optimization

(5) $$\min\sum_{1}^{N_{i}}\left|\tilde{f_{j}}-f_{j}\left(\alpha_{k},\gamma_{m}\right)\right|,$$

where $f_{jkm}$ represents the calibrated *j*-th segment of the measured profile at a given cross section with yaw and pitch angles adjusted by $\gamma_{m}$ and $\alpha_{k}$, $\tilde{f_{j}}$, is the ideal involute profile with parameters found via a designed optical experiment, obtained based on photogrammetry of the target gear in Section 4.1, constructed based on the methods of drawing involute profiles described in [6,20], and $N_{i}$ is the total number of sampled points along the rotational axis of the gear using the *i*-th LTS.

**Figure 3:**
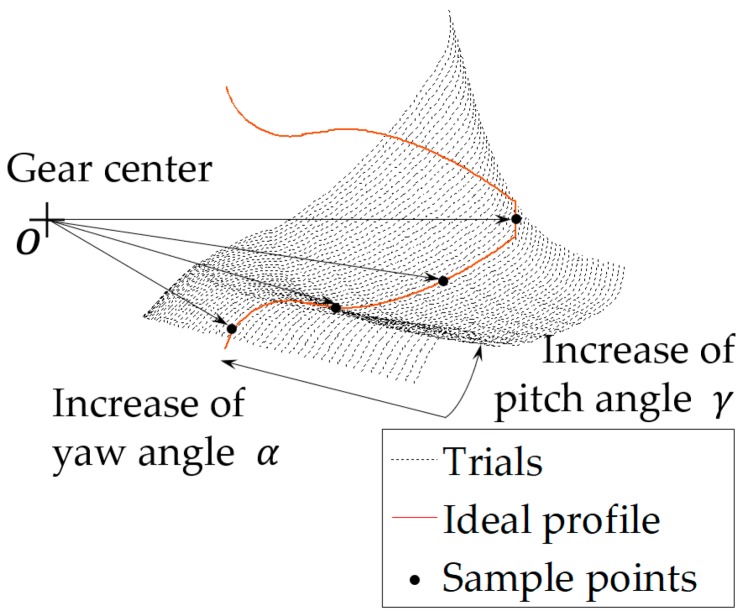
Illustration of the optimization process, note the direction of profile trials versus the increase of the pitch angle α and the yaw angle γ of the LTS mounting orientation. A mesh size of 50 × 60 of the pitch and yaw angles pairs is trialed within the (−10,10) bounds. The absolute radial error between the ideal and trial profiles are compared at the sampled points in a polar coordinate system with the origin identical to that of the gear center.

An illustration of the grid search process [21] to find $\alpha_{k}$ and $\gamma_{m}$ is demonstrated in Figure 3. Before the optimization, an ideal gear tooth profile is generated based on the optical measurement of the one end of the gear. Since the general orientation of the LTS’s to the gear rotation axis before the experiment has been adjusted to near ideal, the deviations of the pitch and yaw angles can be assumed to be small. In this case, the lower and upper bounds for $\alpha_{k}$ and $\gamma_{m}$ are defined as (−10,10) degrees. During the optimization, first a trial involute profile (half of a profile, as shown in Figure 3, due to a single LTS only measures half of the tooth) is constructed by solving the Equations (1) through (4) using the LTS data based on a pair of pitch ($\alpha_{k}$) and yaw ($\gamma_{m}$) angle values within their bounds. Then, the absolute deviation of the trial profile from the ideal is obtained using Equation (4). A subroutine records the absolute deviation at the given pitch and yaw angles. Finally, by iterating through the bounds of $\alpha_{k}$ and $\gamma_{m}$, the deviation between a trial profile and the ideal can be calculated, and the minimum deviation is deemed as the optimized results.

### 2.4. Homogeneous Transformation $H_{0}^{1}$

The gear axis distortion due to mounting error also needs to be calibrated to correctly represent the target point (i.e., $p_{O_{1}O_{2}}^{1}$) on the gear surface. The following homogeneous transformation is performed to represent the desired vector (${\vec{p}}_{O_{1}O_{2}}$) in the *o*_1_*x*_1_*y*_1_*z*_1_ reference frame:

(6) $${\vec{P^{1}}}_{O_{1}O_{2}}=H_{0}^{1}{\vec{P^{0}}}_{O_{1}O_{2}},$$

where $\vec{P}={\left[{\vec{p}}^{T},1\right]}^{T}$ is the homogeneous representation of the vector $\vec{p}$, and $H_{0}^{1}$ is the homogeneous transformation matrix transforming a vector from *o*_0_*x*_0_*y*_0_*z*_0_ to *o*_1_*x*_1_*y*_1_*z*_1_ reference frame, and is defined as

(7) $$H_{0}^{1}=\left[\begin{array}{cc} R_{0}^{1} & \vec{d_{0}^{1}}\\ 0 & 1 \end{array}\right]$$

where $R_{0}^{1}\in {\mathbb{R}}^{3\times 3}$ is the rotation matrix, $\vec{d_{0}^{1}}\in {\mathbb{R}}^{3\times 1}$ is the translation vector from *o*_0_*x*_0_*y*_0_*z*_0_ to *o*_1_*x*_1_*y*_1_*z*_1_ reference frame. Since $R_{0}^{1}$ and $\vec{d_{0}^{1}}$ are dependent upon the location where the LTS stops along the gear’s rotational axis, they are determined experimentally, the design of the experiment is demonstrated in Section 3.2.

According to the proposed measurement setup in Figure 2, the *x*_1_ axis is coincident with the rotational axis of the gear, thus only the rotation about *y*_1_ axis and *z*_1_ axis are considered:

(8) $$R_{0}^{1}=\left[\begin{array}{ccc} \begin{array}{c} \cos\gamma_{1}\cos\beta_{1}\\ \sin\gamma_{1}\cos\beta_{1}\\ -\sin\beta_{1} \end{array} & \begin{array}{c} -\sin\gamma_{1}\\ \cos\gamma_{1}\\ 0 \end{array} & \begin{array}{c} \cos\gamma_{1}\sin\beta_{1}\\ \sin\gamma_{1}\sin\beta_{1}\\ \cos\beta_{1} \end{array} \end{array}\right],$$

where $\gamma_{1}$ and $\beta_{1}$ are the yaw and roll angle from *o*_0_*x*_0_*y*_0_*z*_0_ to *o*_1_*x*_1_*y*_1_*z*_1_ reference frame, and the translation vector from the measurement reference frame to the gear reference frame can be obtained as

(9) $$\vec{d_{0}^{1}}=-{\vec{p^{0}}}_{O_{0}O_{1}},$$

## 3. Experimental Design

### 3.1. Optical Measurement Testbed

**Figure 4:**
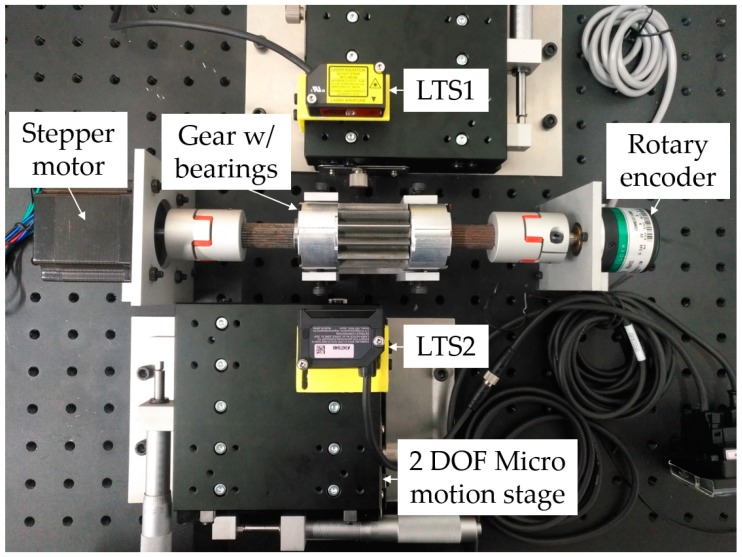
A photo of the experimental setup. Note the application of the twin opposing LTS’s comes from the optical requirement that the incident light beam should be near normal (±12.5°) to the target surface, otherwise the reflection light may not be strong enough to be registered by the receiver on the LTS. Thus, the LTS is placed higher than the gear axis in the *z*_0_ direction. As a result, one half of the gear tooth incline angle satisfies the incident light beam requirement while the other does not. So, to measure the complete gear profile, two opposing LTS’s are used.

A low-cost prototype testbed for noncontact tooth profile measurement is fabricated in lab and assembled with off-the-shelf components as shown in Figure 4. The key geometry parameters are measured and recorded in Table 1. The target gear is disassembled from a working external gear pump, then mounted onto a shaft, which is supported by a pair of tightly toleranced journal bearings installed on both shoulders. One end of the shaft is connected to a rotary encoder (2400 pulses per revolution) and the other to a stepper motor. Both connections are made via flexible shaft couplers. Two opposing laser triangulation sensors are mounted on top of two micro motion stages via 3D printed L-brackets. The micro motion stage is capable of translational movement in both the *x* and *y* directions in a cartesian coordinate system, driven manually with a pair of micrometer screw gauge. The displacement precision is 1 μm. The *z* direction or the altitude of the sensor can be adjusted by a pair of screws on the L-brackets. The stepper motor is controlled using a micro controller unit (MCU, model: Arduino UNO, Arduino AG, Italy), and the analogue voltage signals of the LTS’s and the rotary encoder are acquired by a data acquisition system (DAQ, model: NI USB6002, National Instruments, Austin, TX, USA).

### 3.2. Test Procedures

The three goals of the experimental study are: (1) measurement of the displacement data from the gear tooth surface by LTS, (2) extraction of the target gear profile information based on photogrammetry as the ideal reference, and (3) identification of the spatial distortion of the rotational axis of the gear shaft. The measurement of the tooth displacement data is the basis for reconstruction of the tooth profile, comparable to the traditional way using a mechanical probe. During the experiment, the stepper motor would drive the gear shaft to rotate in constant angular velocity, the micro motion stages are manually adjusted to translate in a line parallel to the gear’s rotational axis and stop at the 13 set sample intervals evenly spaced from 0 mm to 36 mm (total length of the gear). At one sample position, the data acquisition system records the LTS data containing two full revolutions, once finished, the LTS’s are carried by the stage to the next set point. For comparison, the reference tooth profiles from the actual gear geometry are obtained using photogrammetry. An image of the side land of the spur gear is obtained when the focal plane of the camera is coincident with the side land of the gear. In later sections, image processing techniques are applied to the captured image for tooth profile identification. In the last experiment, to identify the relative deviation between the global (*o*_0_*x*_0_*y*_0_*z*_0_) and the gear (*o*_1_*x*_1_*y*_1_*z*_1_) reference frames (i.e., $\gamma_{1}$ and $\beta_{1}$ in $R_{0}^{1}$, and the components of ${\vec{p}}_{O_{0}O_{1}}$), one side of the L-bracket is adjusted such that the LTS is positioned in alignment with the *z* axis of the testbed right above the gear, which allows measurement of the vertical displacement of the gear. Then the gear shaft is locked to one angular position, while the two LTS scan along the set sample locations to obtain the horizontal and vertical deviation data.

In the experiment, synchronous real-time acquisition of gear cross section displacement data from both sides of the gear and the angular position of the gear shaft from the rotary encoder. The programming of the MCU allows the stepper motor to rotate at steady 30 rpm or stopped on demand based on the aforementioned test procedures. The acquisition starts after the system is warmed up for 10 minutes to avoid temperature drift. The sampling frequency is 10 kHz. The parameters of the LTS can be found in Table 2.

## 4. Results and Discussions

### 4.1. Preprocess

According to the procedures discussed in Section 3.2, before the reconstruction of the gear tooth profile, an ideal profile of the gear needs to be obtained as the first preprocess task. The target spur gear is disassembled from an external gear pump with involute gear profile but parameters unknown. Theoretically, a well-calibrated CMM can provide the reference profile needed. However, dimension of the target gear in this work is too small (in this case, the outer diameter is less than 40 mm) for common CMM, and the convex features between teeth interfere with the probe during the measurement of the key dimensions near the fillet. Due to these limitations, photogrammetry is used instead. Though measurement of the feature dimension from a photograph is limited by the accuracy of the optical device and the image processing techniques. However, this work exploited the fact that in gear making industry, the involute profile gears are made to standard sizes, i.e., the pitch number and the number of teeth can only be from a discrete set of values due to the standardization of tooling [20]. Thus, as long as the photogrammetry can distinguish the difference between the two closest dimensioned gears, this method is capable of retrieving the target gear dimensions.

**Figure 5:**
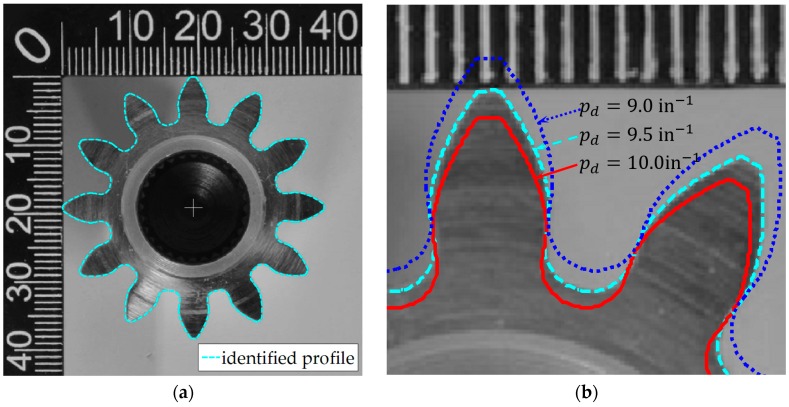
Identified gear profile from an optical image of the gear based on photogrammetry: (**a**) Identified profile and gear center (the cross mark); (**b**) Comparison of the effect on the generated ideal gear profile over three diametral pitch values. By calibrating the pixel width to the reference length on the figure, a conversion coefficient from pixel number to length is found to be 103 pixel/mm.

The image processing technique applied to identify the target gear profile is described as follows. First, a series of morphological processing techniques (i.e., binarizing of the pixel values, closing of holes, and boundary tracing, for details one can refer to [6]) are applied to the original image to locate the rotational center from the dedendum circle (i.e., the dark circular region in Figure 5a with center position identified and marked with a cross). Then the pitch diameter is estimated to be 32 mm based on the distance between the middle point of the tooth height to the center of gear by pixel counting. Using the diametral pitch definition, $p_{d}=\frac{N}{d}$, and substituting in the number of teeth (*N* = 12), the feasible diametral pitch range is found between 9 in^−1^ and 10 in^−1^. The standard values satisfying the range are 9 in^−1^, 9.5 in^−1^, or 10 in^−1^ [22], resulting the pitch diameters to be 33.7 mm, 32.1 mm, or 30.5 mm. The gear profiles based on these diametral pitch values are constructed in Figure 5b. Since the minimum difference is more than 1.6 mm, the resolution of the photo is accurate enough for the determination of the diametral pitch, which is 9.5 in^−1^ in this work. Then in turn, the found diameter info can be used to calibrate the figure dimension, and a 103 pixel/mm conversion coefficient from pixel number to length is found. Comparing to the American Gear Manufacturers Association (AGMA) standard [20], the target gear has shown signs of modification to the standard parameters, which are the rounded corner, addendum and dedendum circle radii. The detailed gear parameters are shown in Table 3. And the optically acquired profile from the flank of the gear is used as the ideal profile (at $x_{1}=0$ mm) in the following sections.

**Figure 6:**
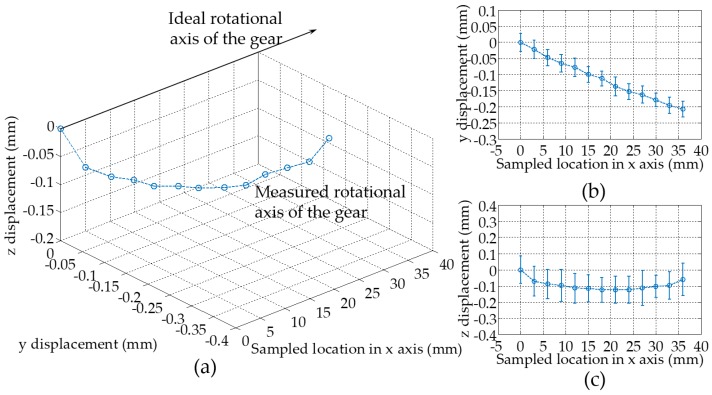
Measured horizontal and vertical gear axis displacement along the *x*_0_ axis: (**a**) 3D view of the displacement variation; (**b**) Vertical displacement variation; and (**c**) Horizontal displacement variation. Note the error bars in (**b**) and (**c**) denote the minimum and maximum deviations of the measurement over 5000 samples based on the combined accuracy of the LTS and data acquisition system (DAQ) systems.

Another task is to determine the relative deviation between the global (*o*_0_*x*_0_*y*_0_*z*_0_) and the gear (*o*_1_*x*_1_*y*_1_*z*_1_) reference frames. The averaged gear axis displacement values in the *y*_0_ and *z*_0_ axes at set positions along the *x*_0_ axis are shown in Figure 6. The measured rotational axis of the gear contains the geometrical tolerance information, i.e., a sum of both the positional error from the micro motion stage and the error from installation of the gear. From Figure 6b, the angular difference between the *z*_1_ and the *z*_0_ axis is found to be 0.3°. In Figure 6c, the vertical displacement plot shows that the majority of the data is parallel to the *x* axis, suggesting the vertical deviation is caused by mounting error near the two extreme positions along the gear axis. Another potential cause due to the gear manufacturing error has been ruled out, since the typical hydrodynamic clearance and the modern manufacturing tolerances for gears are on the order of micro meters in hydraulic systems. The deviation in Figure 6 is one magnitude higher than that of the typical value, which would render the gear pump defective. Yet the gear is disassembled from a working pump, indicating the dominating cause of the rotational axis distortion due to mounting error.

### 4.2. Optimization-Based Tooth Profile Calibration

**Figure 7:**
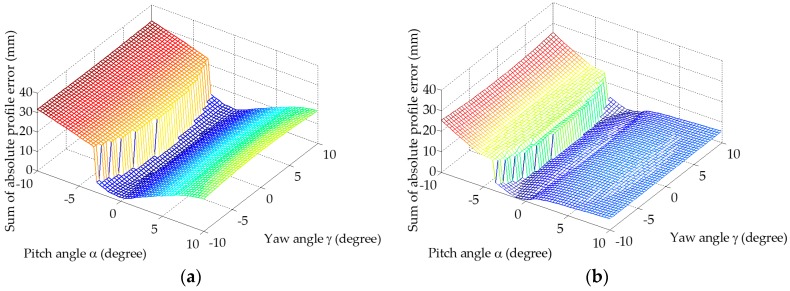
Solution spaces of the optimization to find the yaw and pitch angles: (**a**) Results for the left half of the gear profile with LTS 1; and (**b**) Results for the right half with LTS 2. Note each mesh contains 3000 vertices, a vertex in the plot represents the result of one iteration.

The solution surfaces of the optimization to identify the pitch and yaw angles for both LTS’s are shown in Figure 7. Each vertex in the mesh denotes one trial of the reconstructed profile based on the given pitch and yaw angles. The orientations of the two LTS are different due to individual installation of each on a different micro motion stage supported by an adjustable L-bracket, resulting discrepancies between the two solution spaces in Figure 7a,b. However, the general shapes of the two are similar. For instance, both figures show signs of symmetry about the pitch angle axis when the yaw angle is at 0 degree. This feature suggests that only the magnitude of the yaw angle is important to the sum of the absolute error between the reconstructed and the ideal gear tooth profiles. The optimal LTS pitch and yaw angles for the left and right halves of the test system are (γ=±5.9, α=−0.17) degrees and (γ=±9.2, α=−1.53) degrees, respectively.

### 4.3. Plannar Reconstruction of the Gear Tooth Profile

**Figure 8:**
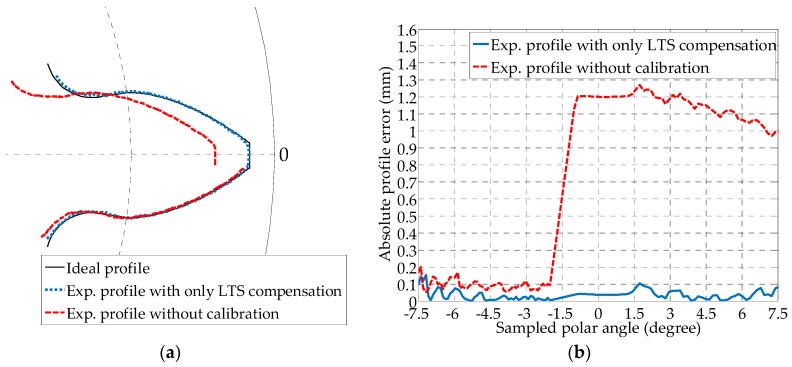
Error comparison between the calibrated gear profile result at *x*_1_ = 0 mm, with yaw and pitch angle compensated from installation error, and the uncalibrated result without spatial error compensation: (**a**) Comparison of reconstructed tooth profiles; and (**b**) Comparison of profile error with respect to the ideal profile.

Before the spatial reconstruction of the profile, the tooth profile at each sampled cross section is processed, and the effects of the proposed calibration on the profile reconstruction accuracy are analyzed in Figure 8. The uncalibrated profile based on Equation (1), the LTS orientation compensated one using Equation (3), and the ideal profiles are compared in a polar coordinate system at $\;x_{1}=0$ mm. Each LTS measures half the profile of a tooth, i.e., from the bottom land through the involute profile to the top land. Applying Equations (1) to (4) to the measured sensor data from one LTS results in half of a tooth profile, whereas by inserting the measurement from the other LTS, the other half can be obtained. Since an overlap exists at the top land of the gear along the addendum between the two calculated profiles, to combine the two halves, a “stitch point” can be selected anywhere from the overlapped region. In this work, the stitch point is consistently selected to be on the intersection point between the right involute and the addendum circle from the left, as shown in Figure 8a. At the origin of the *o*_1_*x*_1_*y*_1_*z*_1_ frame, the axis of the gear has not yet deviated from the *x*_0_ direction of the *o*_0_*x*_0_*y*_0_*z*_0_ frame, in this case $R_{0}^{1}=I\in {\mathbb{R}}^{3\times 3}$ (the identity matrix) and $\vec{d_{0}^{1}}=o\in {\mathbb{R}}^{3\times 1}$. So the homogeneous transform matrix becomes $H_{0}^{1}=\left[I\;0;o\;0\right]$. Thus, only the optimized pitch and yaw angles are required for the calibration using Equation (3). From Figure 8a, the calibrated experimental profiles based on the yaw and pitch correction show better alignment with the ideal profile compared to the uncalibrated case. By comparing the profile error in Figure 8b, the calibrated one is also superior in both the variation of the error and the error magnitude. The maximum absolute variation of the calibrated profile error is within 0.1 mm.

**Figure 9:**
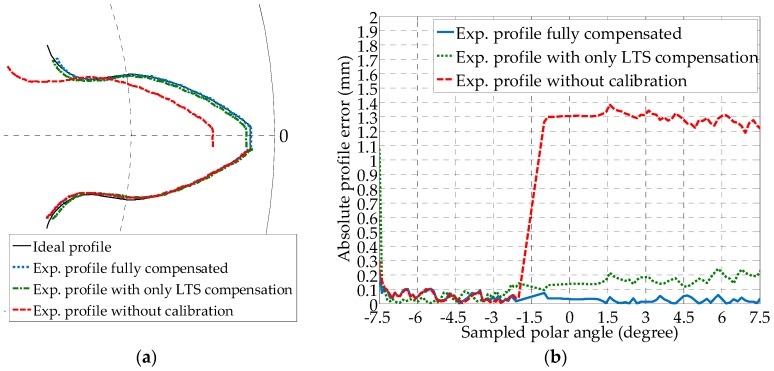
Error comparison between the calibrated gear profile result at *x*_1_ = 35 mm, with only LTS compensation, full calibration with mounting error compensated based on homogeneous transformation, and the uncalibrated result: (**a**) Comparison of reconstructed tooth profiles; and (**b**) Comparison of profile error with respect to the ideal profile.

Effects of the gear axis distortion compensation at the far end of the gear ($x_{1}=35$ mm) are studied in Figure 9. At the opposite end of the gear in the *o*_1_*x*_1_*y*_1_*z*_1_ frame, the axis of the gear has shown significant deviation from the ideal in the *x*_0_ direction of the *o*_0_*x*_0_*y*_0_*z*_0_ frame according to Figure 6b. Based on the measured displacement and rotational angle, the homogeneous transform matrix can be updated at the current axial displacement using Equation (7), in addition to the optimized pitch and yaw angles. In the comparison of the profiles in Figure 9a, the fully compensated profile shows the best alignment with the ideal. Figure 9b confirmed the finding. For the case with the optimized pitch and yaw angle but without axis distortion compensation, the error is over twice that of the fully calibrated one, the magnitude is over 0.2 mm. And the error for the fully compensated profiles is under 0.1 mm, demonstrating the effect of the combined compensation of the axis distortion and the orientation of the LTS.

### 4.4. Spatial Reconstruction of the Gear Tooth Profile

**Figure 10:**
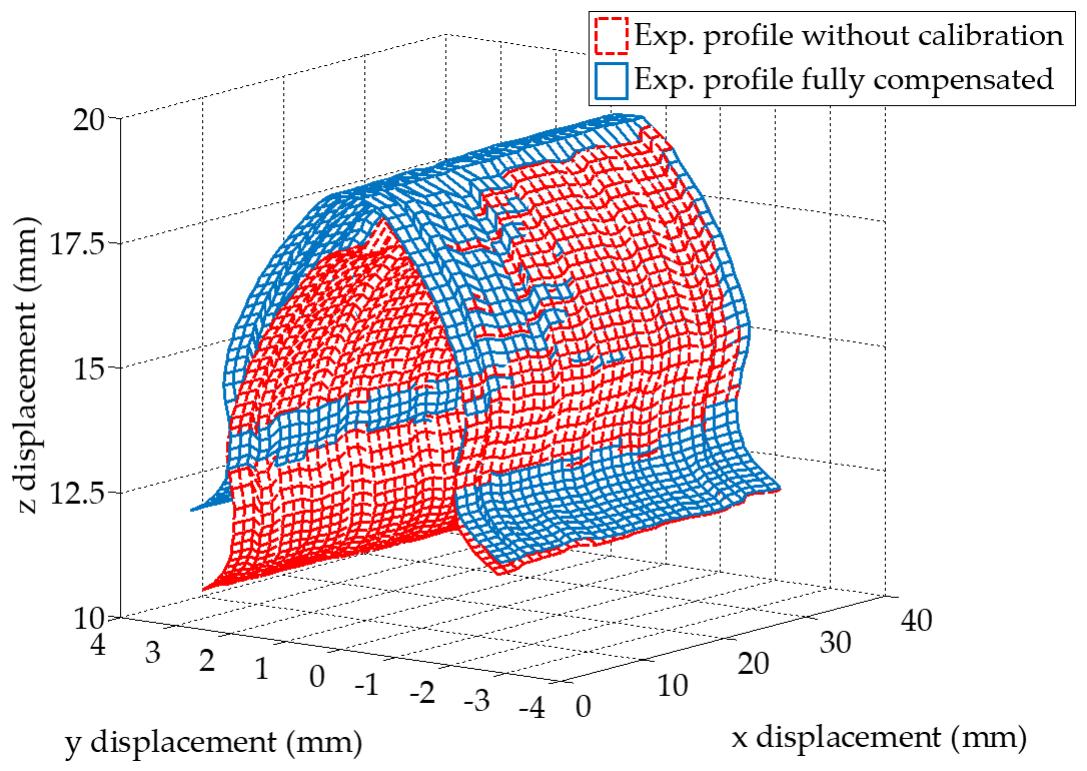
Reconstructed gear profile based on the proposed method. Note the uncalibrated profile is distorted and deformed from the calibrated one. Also, each vertex in the mesh represents one data point. The actual data set is two orders of magnitude denser than that in the plot, the sparsification of data is for clear illustration.

**Figure 11:**
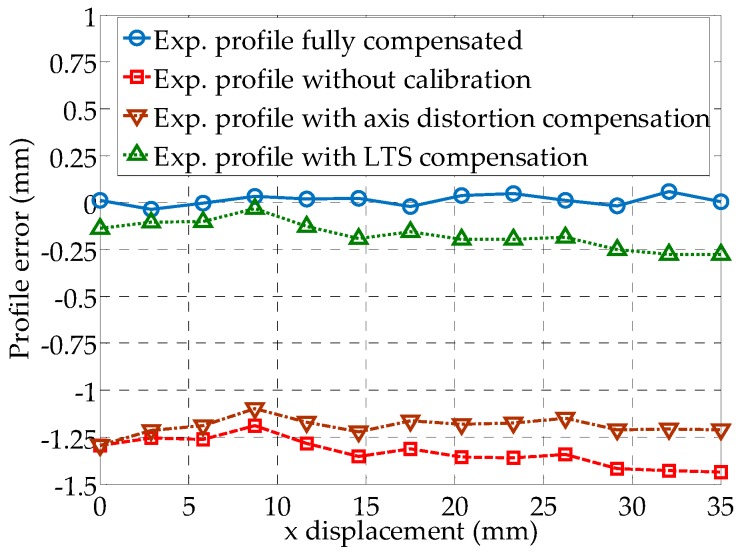
Average profile error trend along the gear rotational axis under four difference compensation settings.

The measured tooth displacement data sets along the gear axis is assembled in 3D with and without geometric compensation by the LTS orientation (Equation (3)) and gear axis distortion (Equation (7)) as shown in Figure 10. It is seen that the calibration of the measurement has significant effect on shape of the spatial gear profile. Also, as the *x*_1_ displacement is increasing, an expected trend is noticed that the uncalibrated profile deviates more with respect to the calibrated one as the sampled location is moved from 0 mm towards the 35 mm in the *x*_1_ axis, supporting the observations in Figure 6b,c. This trend is also seen in the average profile error trend plot along the gear rotational axis under difference compensation settings when compared to the ideal profile optically acquired, as in Figure 11. The profile error of the fully compensated experimental results compared to ideal one is between 3.7 μm and 59 μm, which limiting factor is the accuracy of the LTS. An average of experimental profile error reduction by the compensation of the gear axis distortion, the LTS orientation and the previous two combined are 11%, 87%, and 98%, respectively. The proposed method has shown effectiveness in reducing the geometrical error inherited from the mounting and installation errors.

## 5. Conclusions

Measurement of gears in mobile fluid power systems requires specialized tools. In this work, the working principle of a gear profile measurement apparatus was proposed. The key geometrical models were derived. A testbed was designed and built to measure the gear profile and algorithm given to reconstruct the tooth profile in the 3D space. An optimization was performed to determine the orientation error of the two LTS’s. In this way, the measured tooth profile can be calibrated by compensating the sensors’ pitch and yaw angles. Preliminary results showed that the relative rotation matrix from the orientation of the LTS to the reference frame of the gear was found influenced by the installation precision and dimensional tolerance of both the gear shaft and the micro motion platform along the gear’s rotational axis. The proposed apparatus is capable of measuring the tooth profile, and the geometrical model can well predict the mounting error and orientation disturbances. Compared to the uncalibrated tooth profile, the calibrated results can reduce the geometrical error by up to 98%. In the future, LTS with higher resolution will be implement for improved reconstruction accuracy.

## Figures and Tables

**Table 1:** Installation geometrical information of the twin LTS’s.

LTS 1 *x*_0_ Location $p_{O_{0}E,1}^{0}\;(\mathrm{mm})$	LTS 1 *y*_0_ Location $p_{O_{0}F,1}^{0}\;(\mathrm{mm})$	LTS 1 *z*_0_ Location $p_{O_{0}G,1}^{0}\;(\mathrm{mm})$	LTS 2 *x*_0_ Location $p_{O_{0}E,2}^{0}\;(\mathrm{mm})$	LTS 2 *y*_0_ Location $p_{O_{0}F,2}^{0}\;(\mathrm{mm})$	LTS 2 *z*_0_ Location $p_{O_{0}G,2}^{0}\;(\mathrm{mm})$
0	79.9	6.1	0	54.4	7.7

**Table 2:** Main parameters of the LTS.

Measurement Range (mm)	Linearity* (%)	Laser Wavelength (nm)	Beam Diameter (μm)	Response Frequency (kHz)
100 ± 35	±0.1	655	120	0.66

*Note: For the ±0.1% linearity, it suggests the accuracy of the sensor is ±70 μm. However, since the measurement only takes place under 8 mm for the particular gear, in actual test, the LTS has been calibrated between 0 mm and 10 mm to the standard sized blocks. Thus, within the calibrated range, the ±0.1% linearity can result in ±10 μm accuracy. The drawback is the shortened range of measurement.

**Table 3:** Main parameters of the identified spur gear.

Number of Teeth	Pitch Circle Radius (mm)	Addendum Radius (mm)	Dedendum Radius (mm)	Fillet Radius (mm)	Pressure Angle (°)
12	16.0	19.2	12.6	1.33	20

## Appendix A

A pure rotation about the fixed reference frame can be stepped by a series of rotations about one axis at a time [23]. Based on the reference frame defined in Section 2.1, the matrix describing the yaw, roll and pitch about the *x*, *y* and *z* axes is:

$$R_{t}=R_{z,\gamma }R_{y,\beta }R_{x,\alpha }=\left[\begin{array}{ccc} \begin{array}{c} \cos\gamma \\ \sin\gamma \\ 0 \end{array} & \begin{array}{c} -\sin\gamma \\ \cos\gamma \\ 0 \end{array} & \begin{array}{c} 0\\ 0\\ 1 \end{array} \end{array}\right]\left[\begin{array}{ccc} \begin{array}{c} \cos\beta \\ 0\\ -\sin\beta \end{array} & \begin{array}{c} 0\\ 1\\ 0 \end{array} & \begin{array}{c} \sin\beta \\ 0\\ \cos\beta \end{array} \end{array}\right]\left[\begin{array}{ccc} \begin{array}{c} 1\\ 0\\ 0 \end{array} & \begin{array}{c} 0\\ \cos\alpha \\ \sin\alpha \end{array} & \begin{array}{c} 0\\ -\sin\alpha \\ \cos\alpha \end{array} \end{array}\right]$$

Since the rolling of the LTS does not affect measurement (as long as the laser beam is not block in any way), the roll matrix is assumed to be identity. Through matrix multiplication, Equation (4) is arrived. Similarly, the rotation matrix from the global to the gear frame ($R_{0}^{1}$) can be found by matrix multiplication, where $R_{x,\alpha }=I\in {\mathbb{R}}^{3\times 3}$.
